# Acute effects of citrulline malate and L-arginine, alone and in combination, on anaerobic performance indicators in highly trained taekwondo athletes

**DOI:** 10.3389/fnut.2026.1788549

**Published:** 2026-03-25

**Authors:** Halil Uçar, Murat Ozan, Yusuf Buzdağlı, Adem Savaş

**Affiliations:** 1 Department of Physical Education and Sports, Faculty of Sport Sciences, Inonu University, Malatya, Türkiye; 2 Department of Physical Education and Sports, Institute of Winter Sports and Sports Sciences, Atatürk University, Erzurum, Türkiye; 3 Department of Coaching Education, Faculty of Sport Sciences, Atatürk University, Erzurum, Türkiye; 4 Department of Coaching Education, Faculty of Sport Sciences, Erzurum Technical University, Erzurum, Türkiye; 5 Department of Food Engineering, Giresun University, Giresun, Türkiye

**Keywords:** agility, citrulline-malate, CMJ, lactate, L-arginine, Wingate

## Abstract

**Purpose:**

This study aimed to examine the acute effects of citrulline malate (CITMAL), L-arginine (L-ARG), and their combined supplementation on anaerobic power output during a standardized Wingate test, as well as on post-exercise agility and countermovement jump performance in highly trained taekwondo athletes.

**Methods:**

The study was designed as a double-blind, randomized, crossover trial. Sixteen highly trained male taekwondo athletes ( ≥ 18 years) completed four experimental conditions— L-ARG (6 g), CITMAL (8 g), combined L-ARG + CITMAL, and placebo (PLA)—administered in powder form 1 h before exercise, with each trial separated by 72 h. Anaerobic power was assessed using a standardized Wingate test, followed by post-exercise agility and countermovement jump evaluations. Data were analyzed using repeated-measures analysis of variance with appropriate *post hoc* adjustments, and statistical significance was set at *P* < 0.05.

**Results:**

Compared with placebo, combined CITMAL + L-ARG supplementation significantly increased peak power output (747.85 ± 115.61 vs. 631.61 ± 108.40 W, *P* < 0.001) and relative peak power (11.72 ± 1.35 vs. 9.94 ± 1.16 W⋅kg^–1^, *P* < 0.001). Agility performance improved by approximately 10.5% (16.26 ± 0.81 vs. 18.17 ± 0.69 s, *P* < 0.001), while CMJ height increased by 22.9% (32.36 ± 3.43 vs. 26.34 ± 2.75 cm, *P* < 0.001). Post-exercise blood lactate concentrations were also significantly lower following combined supplementation (12.93 ± 1.12 vs. 15.20 ± 1.23 mmol⋅L^–1^, *P* < 0.001).

**Conclusion:**

The findings of this study indicate that combined L-ARG and CITMAL supplementation is associated with improvements in selected anaerobic performance, agility, and countermovement jump outcomes in highly trained taekwondo athletes. Compared with PLA and single-supplement conditions, the combined supplementation elicited more favorable responses in key Wingate-derived power variables and post-exercise performance measures. Nevertheless, these results should be interpreted cautiously, and further well-controlled studies are required to confirm the observed effects and to clarify the underlying physiological mechanisms.

## 1 Introduction

Taekwondo is a full-contact martial art in which successful performance is largely determined by the execution of high-force kicking techniques directed to the trunk protector or head of the opponent (1, 2). Competitive taekwondo performance is characterized by repeated bouts of short-duration, high-intensity actions interspersed with brief recovery periods, requiring contributions from both anaerobic and aerobic energy systems (3). Official taekwondo matches consist of three rounds of 2 min, during which explosive actions such as rapid kicking sequences, accelerations, and defensive maneuvers are repeatedly performed, resulting in a predominance of anaerobic energy demands.

Given the combative and intermittent nature of the sport, taekwondo athletes are required to execute technical actions with high precision under time pressure, often while rapidly changing direction and responding to unpredictable stimuli. Consequently, agility—defined as the ability to rapidly change direction while maintaining postural control—and the capacity to perform repeated high-intensity actions are considered key physical components of taekwondo performance. Accordingly, taekwondo training programs frequently emphasize exercises targeting agility, rapid force production, and short-duration high-intensity actions performed at a high technical frequency (4, 5).

The use of dietary supplements has been widely investigated as a potential approach to support anaerobic performance and sport-specific physical attributes such as agility and speed. In recent years, particular attention has been given to supplements with vasodilatory properties, as their use among athletes has become increasingly prevalent (6, 7). Nitric oxide (NO) is a key signaling molecule involved in the regulation of blood flow and vascular function during exercise and plays an important role in skeletal muscle perfusion and blood pressure regulation (8, 9).

During physical activity, NO-mediated vasodilation may facilitate blood flow to active skeletal muscle, thereby supporting oxygen delivery and metabolic regulation, which can influence exercise-related performance outcomes (10). Endogenous NO production occurs primarily through the conversion of L-arginine (L-ARG) and L-citrulline via nitric oxide synthase, a calcium-dependent enzyme, while dietary nitrate also contributes to NO availability through alternative metabolic pathways. Within this physiological framework, nutritional strategies targeting NO-related pathways have been explored for their potential relevance to high-intensity exercise performance.

Among dietary supplements investigated for their potential role in sports performance, L-arginine (L-ARG) has received considerable attention due to its contribution to nitric oxide (NO) synthesis and bioavailability. Through its involvement in NO-related pathways, L-ARG supplementation has been associated with changes in vascular function and blood flow, which may influence exercise-related physiological responses (6, 11–13). Accordingly, several studies have examined the effects of L-ARG supplementation on selected performance outcomes, with some reporting improvements in athletic performance and anaerobic capacity under specific experimental conditions (14–16).

Despite this growing body of literature, the majority of existing research has focused on endurance-oriented or mixed-exercise models. In contrast, combat sports such as taekwondo are characterized by repeated bouts of short-duration, high-intensity actions with brief recovery periods, placing substantial demands on anaerobic energy pathways. Consequently, relatively few studies have specifically examined the effects of L-ARG supplementation on anaerobic performance and post-exercise recovery in combat sport athletes. Addressing this gap may help to clarify the context-specific role of L-ARG supplementation within high-intensity, intermittent sport settings.

Citrulline, as an endogenous precursor of L-arginine (L-ARG), has been proposed as an effective strategy to elevate plasma L-ARG availability and thereby support nitric oxide (NO)–related pathways (17). Endogenous NO synthesis occurs through the conversion of L-ARG into NO and L-citrulline via nitric oxide synthase (NOS) enzymes (18). Following its synthesis, NO is involved in the regulation of vascular tone by promoting smooth muscle relaxation through cyclic guanosine monophosphate (cGMP)–dependent mechanisms (19, 20).

Citrulline is commonly ingested in the form of citrulline malate (CITMAL), in which malate—an intermediate of the tricarboxylic acid cycle—has been suggested to support oxidative metabolism and ATP production under certain conditions (21). Through its proposed influence on NO-related pathways, CITMAL supplementation may contribute to vascular function and blood flow regulation, which can affect oxygen delivery to active tissues, including skeletal muscle and the central nervous system. In addition, NO-related signaling has been linked to neuromuscular communication processes, potentially influencing performance-related outcomes via modulation of neurotransmitter-mediated pathways. Collectively, these mechanisms highlight the physiological basis through which citrulline supplementation has been investigated in exercise performance contexts, while acknowledging that direct mechanistic effects remain dependent on experimental verification.

Within this context, CITMAL supplementation has been extensively investigated in relation to exercise performance, particularly during high-intensity and endurance-based activities. Several studies have examined the effects of CITMAL supplementation on aerobic and anaerobic exercise performance, as well as post-exercise recovery, using a range of dosages and experimental protocols (22–25). However, the findings across these studies remain inconsistent, and conclusive evidence supporting clear performance or recovery benefits has yet to be established. Such discrepancies may be related to differences in study design, participant training status, supplementation strategies, and selected outcome measures.

In addition, despite the shared involvement of L-ARG and citrulline in nitric oxide–related pathways, relatively few studies have explored the combined supplementation of L-ARG and CITMAL. This gap in the literature highlights the need for further investigation into the acute effects of combined CITMAL and L-ARG supplementation on performance-related outcomes, particularly within high-intensity, intermittent sport contexts.

Anaerobic power, agility, and jump performance are widely recognized as key performance-related attributes in taekwondo. However, the acute effects of L-ARG, CITMAL, and their combined supplementation on these performance variables—particularly when assessed following a standardized high-intensity exercise bout—remain insufficiently understood.

Therefore, the primary aims of this study were (a) to examine the acute effects of L-ARG, CITMAL, and their combined supplementation on anaerobic power output during a standardized Wingate anaerobic test, and (b) to evaluate changes in post-exercise agility and countermovement jump (CMJ) performance under each supplementation condition in highly trained taekwondo athletes.

## 2 Materials and methods

### 2.1 Study design

This study was conducted using a double-blind, randomized, counterbalanced crossover design. All participants completed four experimental conditions (PLA, CITMAL, L-ARG and combined CITMAL + L-ARG), with the order of conditions randomized and counterbalanced across participants. A balanced Latin square design was used to generate four treatment sequences (PLA –CITMAL – –L-ARG - CITMAL + L-ARG; CITMAL – L-ARG – CITMAL + L-ARG - PLA; L-ARG – CITMAL + L-ARG – PLA – CITMAL; CITMAL + L-ARG – PLA – CITMAL -L-ARG), ensuring that each condition appeared equally often in each testing period.

The randomization sequence was generated using a computer-based random number generator implemented in Microsoft Excel by an independent laboratory staff member. The same staff member assigned participants to the experimental conditions according to this sequence.

Participant enrollment and baseline assessments were conducted by a separate independent laboratory staff member who was not involved in supplement preparation, randomization, or allocation. Allocation concealment was ensured using coded supplement containers. The independent laboratory staff members involved in randomization and allocation had no involvement in data collection, performance testing, or statistical analysis. Both participants and investigators remained blinded to the supplementation conditions until completion of data analysis.

**FIGURE 1:**
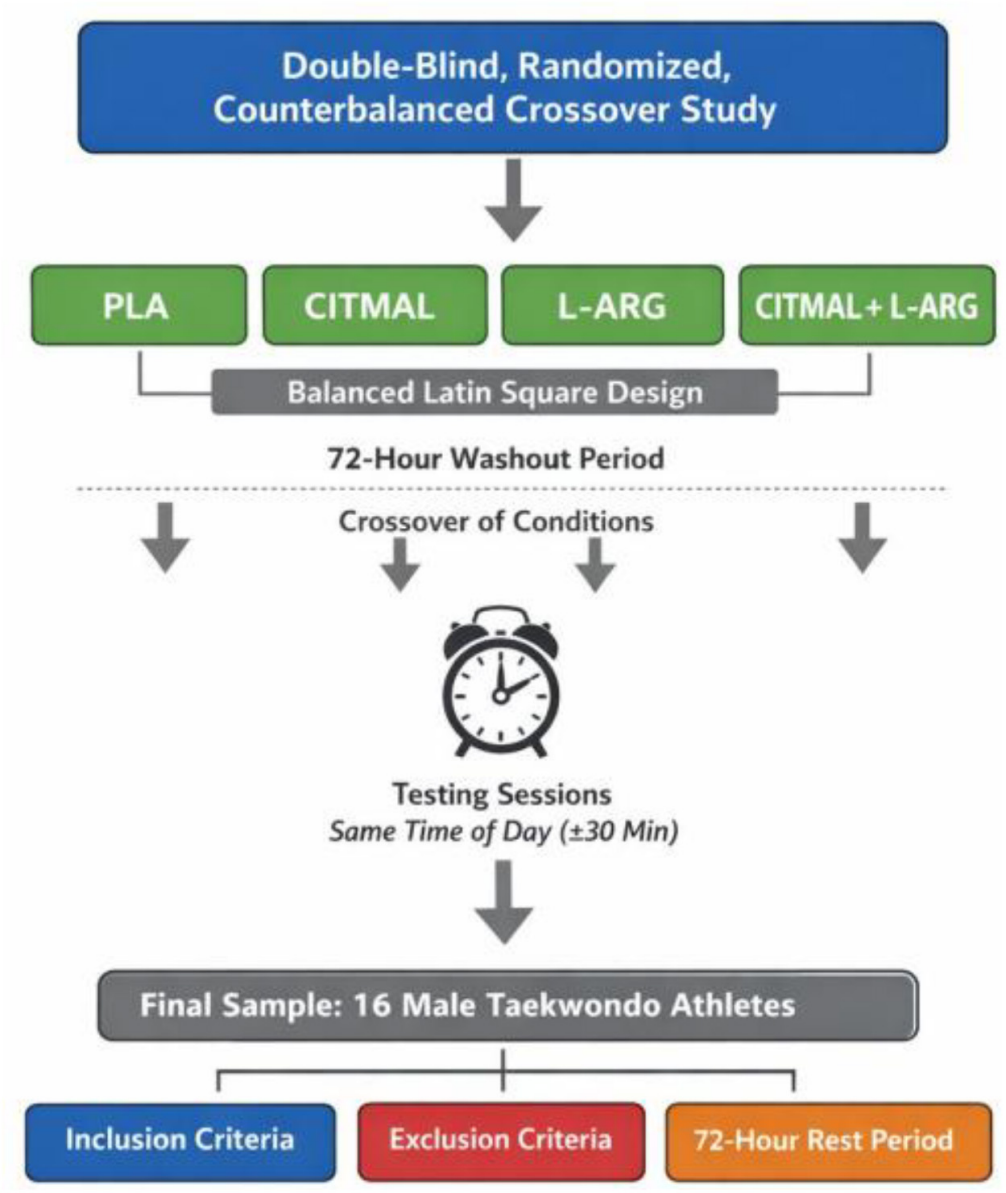
Participant flow diagram and crossover study design.

Each experimental condition was separated by a 72-h washout period to minimize potential carryover effects. To control for circadian rhythm influences, all testing sessions for a given participant were performed at the same time of day (±30 min). Participants completed all experimental sessions following a standardized 72-h rest period and under similar pre-testing conditions. The study design and participant flow are illustrated in Figure 1.

### 2.2 Participants

The study was conducted in Erzurum province and initially included 18 healthy, male taekwondo athletes aged 18 years or older who were engaged in high-level competitive training. Two athletes were excluded from the final analysis due to incomplete performance data resulting from technical recording errors during one experimental session. Missing data occurred in different supplementation conditions and were considered random. Therefore, these participants were excluded from all analyses to preserve the integrity of the crossover design, resulting in a final sample of 16 participants.

Inclusion criteria were: (a) age ≥ 18 years; (b) a minimum of 8 years of competitive taekwondo experience; and (c) achievement of national or international competitive rankings. Exclusion criteria included: (a) use of stimulants, narcotics, or psychoactive substances during the testing or supplementation period; (b) use of dietary supplements, anabolic agents, or other substances known to influence hormonal responses or physical performance within the previous 3 months; and (c) presence of any orthopedic, neurological, cardiovascular, pulmonary, or metabolic condition that could compromise safe participation or test performance. To ensure representation across competitive divisions, the sample was structured to include at least two athletes from each Olympic weight category in taekwondo.

It should be noted that some participants competed in weight-category sports, in which intentional short-term weight reduction is commonly practiced to remain within specific competitive divisions. This may partially explain the low BMI value observed in one participant.

### 2.3 Exercise protocol

To control for potential circadian rhythm effects associated with supplementation, participants completed four experimental sessions, each separated by a 72-h interval and conducted at the same time of day (±30 min) for each individual. In every session, participants performed the same standardized exercise testing sequence. The Wingate anaerobic test was conducted first, followed by the initial blood lactate measurement approximately 30 s post-exercise (±30 s). Two minutes after the Wingate test, participants performed the CMJ test, followed by the Illinois agility test after an additional 2-min interval. A final blood lactate sample was obtained 5 min after completion of the agility test. This standardized testing order was applied consistently across all experimental sessions to allow comparison of post-exercise performance indicators. Seventy-two hours prior to the experimental trials, all athletes completed familiarization sessions and underwent anthropometric assessments. All performance tests used in this study, including the Wingate test, CMJ, and Illinois agility test, have previously demonstrated high test–retest reliability in trained athletic populations, with reported intraclass correlation coefficients exceeding 0.80.

### 2.4 Age, height, and body weight

Age, height, body weight, and body mass index (BMI) were assessed for all participants. Body weight was measured using a calibrated electronic scale (Hüray, Istanbul, Türkiye) with a precision of 0.01 kg, with participants standing barefoot and wearing only shorts. Height was measured with participants barefoot, standing upright with heels together and the head positioned in the Frankfort horizontal plane, using an ultrasonic height measurer (Soehnle Ultrasonic Height Measurer) with a sensitivity of ± 1 mm. Height values were recorded in centimeters. Body mass index was calculated using the standard formula: BMI = body weight (kg)/height^2^ (m^2^).

### 2.5 Supplement protocol

Participants reported to the laboratory 90 min before the start of each experimental session. The supplementation conditions consisted of CITMAL; 8 g; Bulk Supplements, Henderson, NV, United States, L-ARG; 6 g dissolved in 250 mL of water, a combined CITMAL plus L-ARG condition, and a PLA; consisting of 6 g of sucrose dissolved in water, matched in taste and appearance to the active supplements. Although the total mass of the placebo (6 g sucrose) did not fully match that of the combined supplementation condition (14 g), all beverages were prepared in identical volumes and flavored and colored to minimize perceptible differences between conditions. Based on previous literature indicating that circulating levels of citrulline and arginine typically peak approximately 60 min after oral ingestion, all supplements were administered 60 min prior to the exercise protocol.

To maintain the double-blind design, all supplements were dissolved in 250 mL of water and flavored with a neutral citrus aroma. A uniform coloring agent was added to ensure visual similarity across all beverages. Supplement solutions were prepared by an independent researcher not involved in data collection, coded (A, B, C, D), and administered in a randomized order. Both participants and investigators conducting the experimental protocols were blinded to the supplement conditions throughout the study. Although formal assessment of blinding success was not performed, no participant reported perceivable differences in taste, color, or texture between the supplement conditions.

To standardize nutritional status and minimize potential confounding effects, participants were provided with dietary guidelines to maintain similar macronutrient intake (approximately 60% carbohydrates, 30% fat, and 10% protein) during the 72 h preceding each experimental session. Intake of caffeine-containing foods, beverages, and stimulant substances was prohibited for 72 h prior to testing to avoid residual stimulant effects and potential interactions with supplementation. In addition, participants were instructed to refrain from strenuous physical activity and structured training for at least 48 h before each testing session.

#### 2.5.1 Supplement composition, quality control, and adverse event monitoring

The citrulline malate used in this study was provided as a 2:1 formulation, corresponding to approximately 5.3 g of L-citrulline and 2.7 g of malate per 8 g dose, as specified by the manufacturer (Bulk Supplements, Henderson, NV, United States). All supplements were sourced from a supplier operating under Good Manufacturing Practice (GMP) standards and produced according to established quality control procedures.

Throughout the study, participants were monitored for potential adverse events related to supplementation. Gastrointestinal symptoms, including nausea, bloating, and abdominal discomfort, were assessed before, during, and after each experimental session through direct questioning by the research staff. No adverse events or gastrointestinal symptoms were reported by any participant during the study period.

### 2.6 Wingate anaerobic test

The Wingate Anaerobic Test was performed using a mechanically braked cycle ergometer (Monark 894E, Vansbro, Sweden). During the familiarization session, seat and handlebar positions were individually adjusted and standardized for each participant, corresponding to an approximate knee angle of 170–175°, and identical settings were maintained across all experimental sessions. Participants’ feet were secured using toe clips to ensure consistent pedal contact.

Each test began with a standardized 5-min warm-up at 60 W, including two 5-s unloaded sprints performed during the second and third minutes. Following the warm-up, participants completed a 30-s all-out sprint from a stationary start against a resistance set at 7.5% of individual body mass, in accordance with commonly used Wingate testing procedures. Participants were instructed to remain seated, minimize upper-body movement, and maintain continuous maximal pedaling throughout the test. Strong verbal encouragement was provided during each trial to promote maximal effort.

### 2.7 Blood lactate

Capillary blood lactate concentrations were measured using a portable lactate analyzer (Lactate Scout 4, Leipzig, Germany) in accordance with the manufacturer’s instructions. A 5 μL blood sample was obtained from the fingertip using a sterile lancet. Blood lactate measurements were collected at three time points: (1) at rest prior to exercise, (2) approximately 30 s following completion of the Wingate anaerobic test, and (3) 5 min after completion of the agility test.

### 2.8 Counter movement jump

CMJ performance was assessed to evaluate lower-body neuromuscular function. The test was performed using a contact mat system (Optojump, Microgate, Italia) which automatically recorded jump height based on flight time. Participants stood with feet shoulder-width apart and hands placed on the hips to minimize arm swing. From an upright standing position, participants performed a rapid downward movement followed immediately by a maximal vertical jump.

To minimize additional fatigue following the Wingate test, each participant performed a single maximal CMJ trial, which was conducted 2 min after completion of the Wingate anaerobic test. Jump height from this single trial was used for subsequent analysis.

Prior to the experimental trials, all participants completed familiarization sessions for the CMJ test to minimize learning effects. The Optojump system has demonstrated high reliability in trained athletes (ICC > 0.90) in previous studies. A single maximal attempt was used to reduce cumulative fatigue following the Wingate test and to reflect acute post-exercise neuromuscular performance under standardized conditions.

### 2.9 Agility (Illinois) test

Agility performance was assessed using a modified Illinois agility test designed to evaluate rapid directional changes following high-intensity exercise. The test course was set up on a flat indoor surface and consisted of a 10 × 5 m rectangular area with cones placed at each corner and four additional cones positioned along the center line at 3.3 m intervals to form a slalom section. Participants began the test from a standing start behind the starting line and completed the course as quickly as possible while following the predefined running pattern.

Running time was recorded using a dual-beam photoelectric timing system (Tümer Elektronik Ltd., Ankara, Turkey) with a measurement accuracy of 0.01 s. Each participant performed a single maximal trial, which was conducted 4 min after completion of the Wingate anaerobic test. The total completion time was recorded in seconds and used for subsequent analysis.

Participants were familiarized with the Illinois agility test during preliminary sessions. Previous studies have reported high test–retest reliability for this protocol (ICC > 0.85). A single maximal trial was performed to limit additional fatigue and preserve the acute testing design.

### 2.10 Statistical and power analysis

All data are presented as mean ± standard deviation (SD). Normality of data distribution was assessed using the Shapiro–Wilk test. Differences between supplementation conditions were analyzed using repeated-measures analysis of variance (RM-ANOVA), with supplementation condition (PLA, CITMAL, L-ARG and combined CITMAL + L-ARG) as the within-subject factor. When the assumption of sphericity was violated, as indicated by Mauchly’s test, the Greenhouse–Geisser correction was applied. Significant main effects were further explored using Bonferroni-adjusted pairwise comparisons. Effect sizes were calculated using partial eta squared (ηp^2^). Statistical significance was set at *P* < 0.05. All statistical analyses were performed using IBM SPSS Statistics for Windows, Version 21.0 (IBM Corp., Armonk, NY, United States).

An a priori power analysis was conducted using G*Power (version 3.1) to estimate the required sample size for the primary outcome variable, peak power output derived from the Wingate anaerobic test (26). Based on a repeated-measures ANOVA design with four conditions, an assumed medium effect size (*f* = 0.25), an alpha level of 0.05, and a desired statistical power of 0.80, the minimum required sample size was calculated to be 15 participants.

To evaluate potential sequence, period, and carryover effects, an additional repeated-measures ANOVA including sequence and period as fixed factors was performed.

## 3 Results

Participant characteristics and performance outcomes are presented in Tables 1–4. Detailed results for the Wingate anaerobic test, agility performance, blood lactate responses, and CMJ height are shown in the respective tables. The effects of PLA, L-ARG, CITMAL, and L-ARG + CITMAL supplementation on anaerobic performance outcomes were evaluated. Primary outcome measures derived from the Wingate anaerobic test are presented first, followed by post-exercise CMJ performance, agility test results, and blood lactate responses. All results are reported as mean ± standard deviation, and statistically significant differences between conditions are indicated where applicable.

**TABLE 1:** Characteristics of participants.

Variable	Minimum	Maximum	Mean ± SD
Age (years)	18.00	29.00	19.93 ± 2.81
Height (cm)	168.00	187.00	178.50 ± 5.80
Body Weight (kg)	53.70	79.50	63.83 ± 7.82
BMI (kg⋅m^–2^)	15.60	24.60	20.04 ± 2.53

**TABLE 2:** Wingate anaerobic test outcomes under different supplementation conditions.

Variable	PLA Mean ± SD	L-ARG Mean ± SD	CITMAL Mean ± SD	L-ARG*CITMAL Mean ± SD	*F*	*P*	η _*p*_^2^
Peak power (W)	631.61 ± 108.40	676.71 ± 115.34^a^	692.22 ± 117.68^a^	747.85 ± 115.61^abc^	30.281	**< 0.001**	0.669
Peak power (W⋅kg^–1^)	9.94 ± 1.16	10.65 ± 1.29^a^	10.89 ± 1.41^a^	11.72 ± 1.35^abc^	26.616	**< 0.001**	0.640
Mean power (W)	454.98 ± 70.09	458.46 ± 69.62	461.20 ± 63.59	474.38 ± 71.53^ab^	5.993	**0.002**	0.285
Minimum power (W)	244.66 ± 50.40	260.25 ± 51.69^a^	275.38 ± 49.68^ab^	293.35 ± 50.83^ab^	19.792	**< 0.001**	0.569

PLA, placebo; L-ARG, L-arginine; CITMAL, citrulline malate. Superscripts indicate significant *post hoc* differences:

^a^vs. PLA;

^b^vs. L-ARG;

^c^vs. CITMAL (Bonferroni-adjusted, *P* < 0.05). Bold values indicate statistically significant results (*p* < 0.05).

**TABLE 3:** Agility performance and blood lactate responses under different supplementation conditions.

Variable	PLA Mean ± SD	L-ARG Mean ± SD	CITMAL Mean ± SD	L-ARG* CITMAL Mean ± SD	*F*	*P*	η _*p*_^2^
Agility time (s)	18.17 ± 0.69	17.27 ± 0.74^a^	16.88 ± 0.86^ab^	16.26 ± 0.81^abc^	60.007	**< 0.001**	0.800
Baseline Lactate (mmol⋅L^–1^)	2.15 ± 0.38	2.16 ± 0.40	2.09 ± 0.33	2.11 ± 0.36	1.791	0.188	0.107
Post-exercise lactate (mmol⋅L^–1^)	15.20 ± 1.23	14.08 ± 1.25^a^	13.21 ± 1.31^ab^	12.93 ± 1.12^ab^	32.838	**< 0.001**	0.686
Lactate at 5 min post-exercise (mmol⋅L^–1^)	11.84 ± 2.07	11.21 ± 2.35^a^	10.96 ± 2.71	9.86 ± 1.99^abc^	16.750	**< 0.001**	0.528

PLA, placebo; L-ARG, L-arginine; CITMAL, citrulline malate. Superscripts indicate significant *post hoc* differences: ^a^vs. PLA; ^b^vs. L-ARG; ^c^vs. CITMAL (Bonferroni-adjusted, *P* < 0.05). Bold values indicate statistically significant results (*p* < 0.05).

**TABLE 4:** CMJ height under different supplementation conditions.

Variable	PLA Mean ± SD	L-ARG Mean ± SD	CITMAL Mean ± SD	L-ARG*CITMAL Mean ± SD	*F*	*P*	η _*p*_^2^
CMJ height (cm)	26.34 ± 2.75	28.79 ± 3.16^a^	30.14 ± 3.50^ab^	32.36 ± 3.43^abc^	48.559	**< 0.001**	0.764

PLA, placebo; L-ARG, L-arginine; CITMAL, citrulline malate. Superscripts indicate significant *post hoc* differences: ^a^vs. PLA; ^b^vs. L-ARG; ^c^vs. CITMAL (Bonferroni-adjusted, *P* < 0.05). Bold value indicate statistically significant results (*p* < 0.05).

Repeated-measures ANOVA revealed a significant main effect of supplementation condition on peak power (W) (*F* = 30.281, *P* < 0.001, ηp^2^ = 0.669), peak power relative to body mass (W⋅kg^–1^) (*F* = 26.616, *P* < 0.001, ηp^2^ = 0.640), mean power (W) (*F* = 5.993, *P* = 0.002, ηp^2^ = 0.285), and minimum power (W) (*F* = 19.792, *P* < 0.001, ηp^2^ = 0.569).

*Post hoc* analyses indicated that peak power (W) and peak power (W⋅kg^–1^) were significantly higher in the combined L-arginine plus citrulline malate condition compared with PLA, L-ARG, and CITMAL alone. Mean power and minimum power were significantly greater following combined supplementation compared with PLA and L-ARG conditions.

To evaluate potential period, sequence, and carryover effects, a repeated-measures ANOVA including sequence as a between-subject factor and period as a within-subject factor was conducted for the primary outcome (peak power).

A significant main effect of period was observed (*F* = 30.51, *P* < 0.001, ηp^2^ = 0.718). No significant sequence effect was detected (*F* = 1.08, *P* = 0.393, ηp^2^ = 0.213), and no significant period × sequence interaction was observed (*F* = 1.04, *P* = 0.430, ηp^2^ = 0.206), indicating no statistically significant evidence of meaningful carryover effects and supporting the adequacy of the 72-h washout period.

Repeated-measures ANOVA revealed a significant main effect of supplementation condition on agility time (s) (*F* = 60.007, *P* < 0.001, ηp^2^ = 0.800), post-exercise blood lactate concentration (mmol⋅L^–1^) (*F* = 32.838, *P* < 0.001, ηp^2^ = 0.686), and blood lactate concentration measured 5 min post-exercise (mmol⋅L^–1^) (*F* = 16.750, *P* < 0.001, ηp^2^ = 0.528).

*Post hoc* analyses indicated that agility time was significantly shorter following combined L-arginine plus citrulline malate supplementation compared with PLA, L-arginine, and citrulline malate alone. In addition, post-exercise and 5-min post-exercise blood lactate concentrations were significantly lower in the combined supplementation condition compared with PLA and L-ARG conditions.

Repeated-measures ANOVA demonstrated a significant main effect of supplementation condition on CMJ height (cm) (*F* = 48.559, *P* < 0.001, ηp^2^ = 0.764). *Post hoc* analyses revealed that CMJ height was significantly greater following combined L-arginine plus citrulline malate supplementation compared with PLA, L-ARG, and CITMAL alone.

No significant sequence, period, or carryover effects were detected for any of the primary outcome variables (all *P* > 0.05).

## 4 Discussion

The present study examined the acute effects of CITMAL, L-ARG, and their combined supplementation on anaerobic performance, agility, and CMJ height in highly trained male taekwondo athletes. The main findings indicate that combined CITMAL and L-ARG supplementation resulted in greater peak power output during the Wingate test, shorter agility time, and higher CMJ height compared with PLA and single-supplement conditions. In addition, modest improvements in selected Wingate performance parameters were observed following CITMAL supplementation alone compared with PLA. Taken together, the observed changes highlight the potential relevance of combined CITMAL and L-ARG supplementation for acute anaerobic performance outcomes within the context of the present experimental model.

These observations are generally consistent with recent literature examining the effects of nitric oxide-related precursor supplementation on exercise performance. Several meta-analyses and systematic reviews have reported that CITMAL supplementation may elicit small-to-moderate improvements in performance outcomes, particularly during high-intensity, short-duration anaerobic exercise tasks (27–29). For example, Vårvik et al. (30) suggested that CITMAL supplementation may contribute to delayed fatigue and improved muscular endurance during repeated high-intensity efforts.

Recent evidence has further supported the potential ergogenic effects of citrulline malate and nitric oxide–related supplementation on high-intensity exercise performance. For instance, Devrim-Lanpir et al. reported significant improvements in CrossFit performance following acute citrulline malate ingestion (28), while Nobari et al. highlighted its role in enhancing anaerobic capacity and recovery through nitric oxide–mediated mechanisms (29). Similarly, Haugen et al. demonstrated that citrulline malate may positively influence power and jumping performance in trained athletes (23). These recent findings strengthen the relevance of the present results within contemporary sports nutrition research.

In addition, previous studies investigating nitrate- or arginine-related supplementation have reported improvements in performance variables associated with rapid force production and change-of-direction tasks, which may partially support the agility improvements observed in the present study (31). González and Trexler (22) further proposed that the combined intake of CITMAL and L-ARG could lead to more pronounced performance-related responses compared with single-supplement strategies, particularly in anaerobic exercise contexts.

Nevertheless, not all studies have demonstrated beneficial effects of CITMAL supplementation (23, 32). Such inconsistencies across the literature may be attributed to differences in exercise modality, training status of participants, supplementation protocols, and outcome measures. Taken together, these findings suggest that the acute performance responses to CITMAL and L-ARG supplementation may be context-dependent, particularly in experimental models involving high-intensity, intermittent exercise (33–40).

From a mechanistic perspective, CITMAL has been proposed to influence exercise performance through nitric oxide (NO)–related pathways, potentially supporting vasodilation, blood flow regulation, and metabolic by-product clearance during high-intensity exercise (24, 41). Similarly, L-ARG serves as a substrate for NO synthesis and may contribute to vascular and metabolic responses under certain conditions (22).

No biomarkers related to nitric oxide metabolism (e.g., NOx, plasma nitrite/nitrate, arginine, or citrulline concentrations) were measured in the present study. Therefore, the current findings cannot provide direct mechanistic evidence regarding nitric oxide-related pathways. Any mechanistic interpretations discussed herein are speculative in nature and are based solely on previous literature rather than on data derived from the present investigation.

A relatively underexplored aspect of the present study is the assessment of agility performance following supplementation. While previous investigations have primarily focused on endurance- or strength-related outcomes, the observed improvement in agility performance represents an important complementary finding. Tan et al. (42) reported that nitrate supplementation may enhance skeletal muscle function, particularly in fast-twitch fibers, thereby supporting performance during high-intensity exercise.

In sports such as taekwondo, where athletes are required to perform rapid directional changes and explosive movements under conditions of fatigue, improvements in agility performance following CITMAL and L-ARG supplementation may hold potential relevance for performance execution. However, these observations should be interpreted within the controlled experimental context of the present study.

Moreover, the improvements observed in CMJ performance following CITMAL and L-ARG supplementation may reflect favorable performance-related responses associated with nitric oxide precursor intake. The CMJ test is widely used as a sensitive indicator of lower-body explosive performance and is commonly employed to monitor neuromuscular status and fatigue-related changes in athletic populations (43). Previous research has suggested that nitric oxide–related supplementation may influence muscle function and fatigue-related processes during high-intensity exercise, potentially supporting repeated force production (44, 45).

Within this framework, the present findings add to the growing body of literature indicating that CITMAL and L-ARG supplementation may be associated with improved performance outcomes in sports characterized by repeated high-intensity efforts, while acknowledging that direct neuromuscular mechanisms were not assessed in the current study.

Despite these promising findings, several limitations should be acknowledged. One important limitation of the present study is the absence of biochemical measurements, such as plasma NOx or circulating amino acid concentrations. Although performance-related improvements were observed, the lack of biochemical data limits the ability to draw direct mechanistic conclusions, and proposed explanations therefore remain speculative (41).

In addition, while Bonferroni corrections were applied for *post hoc* pairwise comparisons, the evaluation of multiple primary outcomes—including several Wingate-derived parameters, agility, CMJ measures, and lactate responses—may increase the overall risk of Type I error. Moreover, the relatively large effect sizes reported should be interpreted with caution in light of the modest sample size (*n* = 16), as small samples have been shown to potentially overestimate true effect magnitudes (46–48).

From a practical standpoint, the findings of the present study indicate that CITMAL and L-ARG supplementation, particularly when administered in combination, may be associated with favorable performance-related responses in sports characterized by high anaerobic demands, agility, and short recovery periods. Within controlled training settings, such supplementation strategies may be considered during phases emphasizing power-oriented performance and recovery-related outcomes. However, individual variability in response, as well as the potential for gastrointestinal discomfort—especially at higher dosages—should be carefully monitored (49). Given the heterogeneity of findings reported across the literature, supplementation practices should be approached with caution and ideally implemented under professional guidance.

Future research should aim to clarify optimal dosing strategies and timing for CITMAL and L-ARG supplementation, incorporate biochemical analyses to verify proposed physiological mechanisms, and expand investigations to include female athletes and a broader range of sporting disciplines. Such approaches would contribute to a more comprehensive understanding of the context-specific effects of nitric oxide precursor supplementation and help define its potential role within evidence-based training and performance research frameworks.

In conclusion, the present study provides preliminary evidence that CITMAL and L-ARG supplementation, particularly when administered in combination, is associated with improvements in selected anaerobic performance, agility, and CMJ outcomes in highly trained taekwondo athletes. Nevertheless, these findings should be interpreted cautiously in light of the study’s methodological limitations. Another limitation of the present study is that CMJ and agility performance were assessed using a single maximal trial per condition. Although participants were familiarized with the testing procedures and the protocols demonstrate high reliability in trained populations, the use of multiple trials may have reduced measurement error.

A major limitation of the present study is that the placebo condition was not fully matched to the combined supplementation condition in terms of total powder mass. Although extensive blinding procedures were implemented and no participant reported perceivable differences in taste, texture, or gastrointestinal sensations, partial unblinding cannot be entirely excluded. In addition, the effectiveness of blinding was not formally assessed using a structured questionnaire, which represents a further limitation.

Although Wingate, CMJ, and agility tests provide objective outcome measures, they require maximal voluntary effort and may therefore be partially influenced by motivation, expectancy, and perceived exertion. Consequently, expectancy-related effects may have contributed to an overestimation of effect sizes. Therefore, the present findings should be interpreted as preliminary evidence.

Importantly, mass-matching the placebo using sucrose may not necessarily provide an inert control, as oral exposure to carbohydrate alone has been shown to acutely enhance power output via proposed central mechanisms (50). Therefore, complete mass-matching using sucrose could have introduced an additional metabolically active confounder.

Further well-controlled research incorporating biochemical measurements, formal blinding assessments, and larger, more diverse samples is required to confirm these observations and to clarify the underlying physiological mechanisms.

Test–retest reliability indices (ICC, CV%, and typical error) for CMJ and the Illinois agility test could not be derived from the present dataset, as only a single maximal trial was performed under each condition. This represents a major methodological limitation, as the magnitude of the observed changes cannot be clearly distinguished from potential measurement error. Accordingly, the improvements observed in CMJ and agility performance should be interpreted cautiously, and may partially reflect random variability rather than true physiological adaptations. Future studies should incorporate multiple trials and formal reliability assessments to strengthen the validity of these outcomes.

## 5 Conclusion

This study demonstrates that acute supplementation with 8 g of CITMAL and 6 g of L-ARG, administered 1 h prior to exercise, is associated with improvements in selected anaerobic performance parameters in highly trained male taekwondo athletes. In particular, the combined supplementation condition elicited greater responses in Wingate-derived power outcomes, agility performance, and CMJ measures compared with PLA and single-supplement conditions.

These findings should be interpreted within the context of the study’s experimental design and measured outcomes, as no direct assessments of energy system contribution, neuromuscular function, or biochemical markers were performed. Accordingly, the observed performance-related responses cannot be directly attributed to specific physiological mechanisms. Future studies incorporating biochemical analyses and larger, more diverse athletic populations are required to confirm these observations and to further clarify the mechanisms underlying the acute effects of CITMAL and L-ARG supplementation. In addition, the absence of test–retest reliability data for CMJ and agility outcomes limits the strength of conclusions regarding these measures.

## Data Availability

The raw data supporting the conclusions of this article will be made available by the authors, without undue reservation.
